# Determinants of Standard Precautions Performance Among Nursing Students in South Korea: A Cross-Sectional Study

**DOI:** 10.3390/healthcare13212803

**Published:** 2025-11-04

**Authors:** Se Gyeong Jeon, Eun Jung Kim

**Affiliations:** 1 Chonnam National University Hospital, 42, Jebong-ro, Dong-gu, Gwangju 61469, Republic of Korea; tprud0621@gmail.com; 2 Department of Nursing, College of Health Sciences, Honam University, Gwangju 62399, Republic of Korea

**Keywords:** professionalism, universal precautions, morals, students, nursing

## Abstract

**Highlights:**

**What are the main findings?**

- Nursing students’ adherence to standard precautions was significantly influenced by moral courage and academic performance.
- Positive correlations were found between performance of standard precautions and nursing professionalism, knowledge, and moral courage.

**What are the implications of the main findings?**

- Educational programs that strengthen ethical competence and moral courage may enhance infection control practices in nursing students.
- Supporting academic achievement alongside ethical and professional development can better prepare students for safe clinical practice.

**Abstract:**

**Background**: The COVID-19 pandemic highlighted the necessity of standard precautions to prevent healthcare-associated infections. Nursing students, due to limited experience and close patient contact, are at high risk of exposure. This study examined the effects of nursing professionalism, knowledge of standard precautions, and moral courage on nursing students’ performance of standard precautions in South Korea. **Methods**: A cross-sectional survey was conducted with 204 nursing students from 10 universities in Gwangju, South Korea. Validated instruments measured professionalism, knowledge, moral courage, and performance. Data were analyzed using *t*-tests, ANOVA, correlations, and regression. **Results**: Standard precautions performance averaged 4.62/5. It was positively correlated with professionalism (r = 0.306), knowledge (r = 0.190), and moral courage (r = 0.399). Regression identified moral courage (β = 0.38, *p* < 0.001) and academic performance (β = 0.18, *p* = 0.007) as significant predictors, explaining 18.1% of variance. **Conclusions**: Moral courage and academic performance significantly influence nursing students’ adherence to standard precautions. Strengthening ethical competence and professional identity in education may enhance safe clinical practice.

## 1. Introduction

In recent years, the increase in global exchanges and the impact of climate change have contributed to the emergence and spread of various infectious diseases, resulting in significant public health crises [1]. The COVID-19 pandemic, in particular, imposed an unprecedented burden on healthcare systems and underscored the critical importance of infection prevention and control [2]. This experience highlighted that infection prevention within healthcare institutions is not merely a recommendation but an essential component of patient safety.

Standard precautions, developed by the U.S. Centers for Disease Control and Prevention (CDC), represent fundamental infection control guidelines that must be followed to prevent exposure to potentially infectious materials such as blood, body fluids, and secretions [3]. Nurses, who interact very closely with patients, play a pivotal role in infection prevention. Adherence to these guidelines significantly reduces hospital-acquired infections and ensures patient safety [4]. Thus, compliance with standard precautions is indispensable for preventing and controlling the spread of infections within healthcare facilities [5].

According to the Korean nursing education curriculum, nursing students are required to engage in clinical practice in preparation for their future professional roles [6]. During clinical training, they spend considerable time in direct contact with patients through tasks such as measuring vital signs, assisting with hygiene, and providing mobility support. However, due to limited clinical experience and insufficient preparedness for safety incidents, nursing students face higher risks of exposure to infectious agents compared with licensed nurses [7]. Despite this, most Korean nursing programs cover standard precautions only partially, mainly through theory-based courses such as fundamentals of nursing, adult nursing, or community health nursing [8]. According to the World Health Organization, patient safety curricula in health-related university programs should include comprehensive training on standard precautions as a core component of infection prevention and control education [9]. In recent years, nursing education in South Korea has undergone significant changes following the COVID-19 pandemic. Studies published between 2023 and 2025 have shown that virtual reality simulation (VRS)-based infection control training significantly improved nursing students’ infection control knowledge, learning engagement, and confidence [10]. Simulation-based COVID-19 patient care training also strengthened students’ ability to apply standard precautions, use personal protective equipment (PPE), and manage patients in isolation areas [11]. Moreover, flipped-learning infection control programs were found to enhance students’ knowledge and self-efficacy, while international comparative studies highlighted that post-pandemic virtual simulation and remote education have played a key role in improving infection control competencies [12]. Therefore, systematic education and practical training on adherence to standard precautions are essential for nursing students during their clinical practice.

Nursing professionalism refers to nurses’ attitudes, perceptions, and beliefs about their profession, encompassing their views on the role and responsibilities of nursing as a professional discipline [13]. Students with a strong sense of professional identity demonstrate more proactive learning behaviors, greater development of professional competencies, and stronger commitment to continuing nursing after graduation [14]. In clinical contexts, professionalism fosters sound decision-making and enhances patient safety practices [15]. Accordingly, cultivating a positive and solid professional identity is vital for delivering high-quality care and advancing the status of nursing as a profession.

Knowledge of standard precautions is also a critical factor influencing compliance with infection control practices [16]. Previous studies have shown that greater knowledge of standard precautions positively impacts their implementation among nurses [17] and nursing students [18]. Higher knowledge correlates with greater adoption of infection-prevention behaviors in clinical practice, highlighting the importance of effective education on infection control.

Moral courage refers to the willingness to act according to one’s ethical principles and values, even when doing so may involve personal risk or negative consequences [19]. Nurses require moral courage not only to provide safe and high-quality care but also to resist unethical practices [20]. It is essential in situations such as protecting patient privacy or caring for patients with contagious diseases [21]. Conversely, insufficient moral courage may foster unethical conduct, reduce the quality of care, and negatively affect patient outcomes [22]. Nursing students, however, often face challenges in demonstrating moral courage due to hierarchical organizational structures, cultural barriers to self-expression, and limited competence compared to experienced professionals [23,24]. Within the Korean clinical practice environment, students frequently find it difficult to make independent judgments and may feel pressured to conform to social norms, even when these conflict with their personal convictions. Recent European studies have reported that graduating nursing students demonstrate high levels of moral courage, although cultural and educational differences between countries influence these levels [25]. In contrast, in Asian contexts such as South Korea, hierarchical structures and collectivist norms may challenge students’ ability to express moral courage in clinical settings. Therefore, moral courage is essential for nursing students to perform standard precautions and ensure safe care, necessitating intentional training and support [26]. Moreover, moral courage also plays a crucial role when nursing students encounter negative role models who are older or more experienced, as it enables them to uphold ethical principles and resist adopting unsafe or unethical practices. This perspective is supported by Hamed et al. [27] who highlighted that exposure to negative role models can hinder adherence to standard precautions and that targeted education and discussion are needed to mitigate such influences.

Based on this background, the present study aimed to examine the effects of nursing professionalism, knowledge of standard precautions, and moral courage on the performance of standard precautions among nursing students. Findings from this study may provide foundational evidence for the development of infection control education programs designed to enhance compliance with standard precautions.

## 2. Methods

### 2.1. Design and Participants

A cross-sectional descriptive survey design was adopted to identify the factors influencing nursing students’ standard precautions performance.

The participants were undergraduate nursing students from 10 universities located in Gwangju City, South Korea. The data were collected between December 2023 and June 2024. Prior to data collection, the purpose and procedures of the study were explained to the supervisors of the participating institutions and institutional consent was obtained.

Convenience sampling was employed to recruit third- and fourth-year students who had been undergone hospital clinical practice. Only participants who understood the purpose of the study and provided written informed consent were included. Students without clinical practice experience were excluded.

The required sample size was calculated using G*Power 3.1.9.7, with a significance level of 0.05, statistical power of 0.90, and effect size of 0.15. The minimum sample size required was 175 participants. Considering the potential dropout rate of approximately 10%, data were collected from 211 participants. After excluding seven incomplete responses, data from 204 participants were included in the final analysis.

### 2.2. Measures

#### 2.2.1. Nursing Professionalism

Nursing professionalism was assessed using a scale originally developed by Yeun et al. [28] for nurses and subsequently revised and adapted for nursing students by Han et al. [29]. This instrument has been widely used in Korea to evaluate nursing professionalism among nursing students, and its reliability and validity have been consistently supported in prior research. The modified tool consists of 18 items across five subdomains: professional self-concept (6 items), social awareness (5 items), professionalism of nursing (3 items), nursing roles (2 items), and autonomy of nursing (2 items). Each item was rated on a 5-point Likert scale ranging from 1 (“strongly disagree”) to 5 (“strongly agree”), with higher scores indicating greater levels of nursing professionalism. Cronbach’s α was 0.92 in Yeun et al. [28] and 0.94 in Han et al. [29]. In the present study, Cronbach’s α was 0.89.

#### 2.2.2. Standard Precaution Knowledge

Standard precaution knowledge was measured using the instrument originally developed by Seo [30] and later revised and supplemented by Baek [31] based on the standard precaution guidelines updated by the Hospital Infection Control Practices Advisory Committee (HICPAC) in 2007. The tool consists of 29 items across seven subdomains: concept of standard precautions (4 items), hand hygiene (4 items), use of personal protective equipment (11 items), environmental management (4 items), safe injection practices (2 items), respiratory hygiene and cough etiquette (2 items), and patient placement (2 items). Each item was answered with “yes,” “no,” or “don’t know.” Correct answers were scored as 1, while incorrect or “don’t know” responses were scored as 0. Higher scores indicated greater knowledge of standard precautions. The Kuder–Richardson Formula 20 (KR-20) coefficient was 0.70 in Baek [31] and 0.48 in the present study, which may reflect participants’ limited clinical exposure as nursing students.

#### 2.2.3. Moral Courage

Moral courage was measured using the Professional Moral Courage Scale (PMCS), originally developed by Sekerka et al. [32] for military personnel and subsequently translated into Korean and adapted for nursing students by Moon et al. [33]. The instrument consists of 12 items in four subdomains: moral agency and execution (6 items), beyond compliance with orders (2 items), enduring threats (2 items), and multiple value considerations (2 items). Each item is rated on a 7-point Likert scale ranging from 1 (“strongly disagree”) to 7 (“strongly agree”), with higher scores indicating greater levels of moral courage. Cronbach’s α was 0.79 in Moon et al. [33] and 0.81 in the present study.

#### 2.2.4. Standard Precautions Performance

Standard precautions performance was measured using the instrument originally developed by Jung [34], which was based on the Centers for Disease Control and Prevention (CDC) standard precautions guidelines revised in 2007, and later modified by Hong [35] to be more suitable for nursing students. This instrument has been widely utilized in studies involving Korean nursing students, and its reliability has been well established. The tool consists of 36 items across eight subdomains: hand hygiene (10 items), use of personal protective equipment (9 items), respiratory hygiene and cough etiquette (3 items), care of medical equipment and supplies (2 items), environmental management (2 items), linen management (2 items), safe injection practices (5 items), and staff safety (3 items). Each item was rated on a 5-point Likert scale ranging from 1 (“never performed”) to 5 (“always performed”), with respondents instructed to select “no experience” for items they had not encountered. Mean scores were calculated after excluding items marked as “no experience,” with higher scores indicating better performance of standard precautions. Cronbach’s α was 0.95 in Hong [35] and 0.94 in the present study.

### 2.3. Statistical Analysis

The data were analyzed using the IBM SPSS statistical program for Windows, version 28 (IBM Corp., Armonk, NY, USA). Descriptive statistics were used to summarize participants’ general characteristics, nursing professionalism, standard precautions knowledge, moral courage, and standard precautions performance.

Differences in standard precautions performance according to participant characteristics were examined using independent *t*-tests and one-way ANOVA, following a test for normality. As the assumption of homogeneity of variances was satisfied, Scheffé’s post hoc test was employed for post hoc analysis, given its conservative nature in controlling Type I error across multiple comparisons. Pearson’s correlation coefficients were calculated to assess the relationships between key variables. Finally, Stepwise multiple regression analysis was conducted to analyze factors influencing nursing students’ standard precautions performance.

### 2.4. Ethical Issue

This study was approved by the Institutional Review Board of the Honam University in South Korea (approval number: 1041223-202311-HR-38). Participants provided informed consent by completing a pre-consent form in the first section of the online survey. The consent form included information about the research purpose, confidentiality of personal information, and the right to withdraw without any prior justifications. The information provided was aimed at making participants aware of their rights and providing them with the opportunity to make informed decisions about participating in the study. Additionally, the use of anonymous responses further ensured confidentiality and privacy.

## 3. Results

### 3.1. Participant’s Characteristics

A total of 204 nursing students participated in the study. Among them, 80.4% were female and 19.6% were male. The mean age of the participants was 23.40 years, with 37.7% aged 23 years or older. Most respondents were in their fourth year of study (67.2%), and 72.1% reported being satisfied with their nursing major. All participants self-reported their grade point averages (GPA), and 50% indicated that their GPA fell within the range of 3.55–3.99. Regarding clinical practice, 67.2% expressed satisfaction with their training experience. During clinical practice, 8.3% reported having sustained a needlestick or other invasive injury, and 6.4% reported exposure to blood or body fluids. However, only 1% of participants who experienced such incidents formally reported them to supervisors or infection control departments (Table 1).

### 3.2. Nursing Students’ Nursing Professionalism, Standard Precautions Knowledge, Moral Courage, and Standard Precautions Performance

The mean score for the perceived level nursing professionalism was 3.87 (±0.52) out of a possible 5 points. The mean score for perceived standard precautions knowledge was 25.42 (±2.15) out of 29 points. The mean score for moral courage was 5.52 (±0.63) on a 7-point Likert scale.

The average standard precautions performance was 4.62 (±0.39). The mean for each sub-domain was as follows: 4.55 (±0.70) for hand hygiene, 4.63 (±0.59) for personal protective equipment (PPE), 4.63 (±0.55) for respiratory etiquette, 4.80 (±0.43) for treatment devices and supplies, 4.61 (±0.59) for environmental management, 4.66 (±0.56) for laundry management, and 4.90 (±0.29) for staff safety (Table 2).

### 3.3. Differences in Standard Precautions Performance by Participant Characteristics

Significant differences in the standard precautions performance were observed according to participants’ experiences. Gender, age, and year of study did not have a statistically significant effect on standard precautions performance levels. However, nursing students who reported higher satisfaction with their major (M = 4.70 ± 0.38) demonstrated significantly higher standard precautions performance compared with those who were dissatisfied (M = 3.41 ± 0.43; F = 5.35, *p* = 0.005, η^2^ = 0.05). In addition, students with a grade point average of 3.50–3.99 (M = 4.73 ± 0.33) scored significantly higher in performance than those with a GPA below 3.50 (M = 4.53 ± 0.46; F = 5.48, *p* = 0.005, η^2^ = 0.06) (Table 3).

### 3.4. Correlation Among by Variables

A correlation analysis was conducted to examine the relationships among nursing professionalism, standard precautions knowledge, moral courage, and standard precautions performance. According to Cohen’s criteria [36], correlation coefficients of 0.10–0.29 were interpreted as weak, 0.30–0.49 as moderate, and ≥0.50 as strong. Standard precautions performance showed a significant moderate positive correlation with nursing professionalism (r = 0.306, *p* < 0.001), a weak positive correlation with standard precautions knowledge (r = 0.190, *p* = 0.006), and a moderate positive correlation with moral courage (r = 0.399, *p* < 0.001). Nursing professionalism was also positively correlated with standard precautions knowledge (r = 0.168, *p* = 0.016) and moral courage (r = 0.513, *p* < 0.001). In addition, a weak positive correlation was found between standard precautions knowledge and moral courage (r = 0.195, *p* = 0.005) (Table 4).

### 3.5. Factor Affecting Standard Precautions Performance

A stepwise multiple regression analysis was performed to identify the factors influencing nursing students’ standard precautions performance. Variables that showed significant correlations—nursing professionalism, standard precautions knowledge, and moral courage—along with general characteristics that demonstrated significant differences, such as satisfaction with the nursing major and GPA, were included in the regression model. Among these, the nominal variables (major satisfaction and GPA) were converted into dummy variables.

The results revealed that moral courage (β = 0.38, *p* < 0.001) and GPA range of 3.50–3.99 (β = 0.18, *p* = 0.007) were significant predictors of standard precautions performance. The adjusted coefficient of determination (Adj. R^2^ = 0.018) indicates that the model explained 18.1% of the variance in performance. The overall regression model was statistically significant (F = 23.48, *p* < 0.001) (Table 5).

## 4. Discussion

This study aimed to examine the level of standard precautions performance among nursing students and to identify the effects of nursing professionalism, standard precautions knowledge, and moral courage on their performance. The overall performance score of nursing students was 4.62 out of 5, which is consistent with previous findings in Korea [15,37]. The consistently high scores may reflect heightened awareness of infection control following outbreaks such as Middle East Respiratory Syndrome (MERS) and COVID-19, which emphasized the importance of infection prevention across healthcare systems.

Among the subdomains, the highest score was observed for staff safety (M = 4.90), which included items such as avoiding needlestick injuries, discarding used needles without recapping, and refraining from bending or breaking needles. This finding was similar to studies conducted among Turkish nursing students [38] and Korean nursing students [39]. In contrast, hand hygiene recorded the lowest score (M = 4.55), consistent with earlier studies [15]. Despite widespread education, barriers such as lack of paper towels, heavy workload, and time constraints have been reported to negatively affect compliance [40]. Some studies have also indicated that increased opportunities for hand hygiene paradoxically reduce compliance [41]. This suggests that further research is needed to examine the relationship between frequency of opportunities and adherence to hand hygiene [42].

Significant differences in performance were observed based on satisfaction with the nursing major and academic achievement. Students who were satisfied with their major demonstrated higher performance scores, confirming that major satisfaction positively influences adherence to standard precautions. This result is consistent with previous research indicating that students with greater satisfaction are more motivated and better prepared for professional roles [43,44]. Therefore, strategies to improve major satisfaction—such as supportive learning environments, mentorship, and student engagement—may enhance compliance with infection control practices.

GPA was also associated with significant differences. Students with mid-range GPAs (3.50–3.99) scored higher than those with GPAs below 3.50. One possible explanation is that students with mid-range GPAs may maintain a more balanced approach to academic and clinical learning compared with high achievers, who may experience stress or perfectionism that hinders practical performance [45]. This interpretation is supported by recent studies showing that nursing students with higher academic achievement often report greater levels of academic burnout and stress, which can interfere with clinical learning and adherence to infection control practices [46]. Because adherence to standard precautions requires not only knowledge but also behavioral adaptation and practical skills, educational programs should emphasize integrated learning that bridges theoretical knowledge and clinical application.

This study also found significant positive correlations between standard precautions performance and nursing professionalism. These findings are consistent with previous research reporting that stronger professional identity improves clinical competence and safe practice behaviors [47]. Thus, fostering nursing professionalism through structured curricula and experiential learning opportunities is crucial for promoting compliance with standard precautions.

Standard precautions knowledge also demonstrated a positive correlation with performance, in line with previous studies conducted among nurses [15], although results among nursing students have been mixed [48]. This suggests that the relationship between knowledge and practice may vary depending on individual experiences and clinical contexts, warranting further replication studies.

Moral courage was strongly associated with adherence to standard precautions, confirming prior findings that greater moral courage leads to safer and higher-quality nursing care [26,49]. Historically, moral courage has been regarded as a core requirement of nursing since Florence Nightingale’s era. It is influenced by organizational culture, hierarchy, religious beliefs, and personal values [50]. While Korean nursing culture remains relatively hierarchical and authority-driven [51], fostering a relationship-oriented environment based on trust and respect may enhance moral courage among students.

Notably, students with higher moral courage and moderate-to-high academic performance demonstrated greater adherence to standard precautions. This underscores the importance of developing both ethical competence and academic support strategies to improve compliance. Educational interventions that strengthen moral courage—alongside programs that promote balanced academic achievement—may enhance nursing students’ ability to adapt to clinical settings and deliver safe, evidence-based care.

In conclusion, this study highlights that moral courage and academic performance are key predictors of standard precautions performance among nursing students. Nursing education programs should incorporate strategies to reinforce professional identity, strengthen moral courage, and support academic achievement. These efforts may contribute to improving compliance with infection prevention guidelines, thereby enhancing nursing students’ preparedness for clinical practice and ultimately ensuring safer patient care.

This study has several limitations. First, participants were recruited from nursing students in Gwangju, which may limit the generalizability of the findings to students in other regions of Korea. Future studies should include nursing students from diverse geographic areas to enhance the representativeness of the results. Second, data were collected through a self-administered questionnaire consisting of multiple items, which may have restricted the accuracy of participants’ responses. Self-report biases, including social desirability bias (the tendency to respond in ways viewed favorably by others) and recall bias (the limitation in accurately recalling past experiences), may also have influenced the findings. Third, the internal consistency of the instrument measuring standard precaution knowledge was moderate (KR-20 = 0.48), which may have affected the reliability of the findings. Fourth, because academic performance was self-reported, future studies should incorporate objective academic data as well as psychological variables such as stress and burnout to validate and extend the present findings. Finally, the results were derived from the cultural and institutional context of South Korea, which may limit their applicability to countries with different healthcare systems or nursing cultures.

Despite these limitations, this study also has important strengths. To the best of our knowledge, it is the first study in Korea to investigate the effects of nursing professionalism, knowledge of standard precautions, and moral courage on nursing students’ performance of standard precautions. The findings provide foundational evidence for developing specialized, culturally tailored educational and training programs aimed at improving nursing students’ adherence to infection-control practices.

## 5. Conclusions

The findings of this study demonstrated significant positive correlations among nursing professionalism, knowledge of standard precautions, moral courage, and the performance of standard precautions. Among these factors, moral courage and academic performance were identified as significant predictors of nursing students’ adherence to standard precautions. These results highlight the need to develop effective educational programs aimed at enhancing nursing students’ performance of standard precautions.

Improving adherence to standard precautions during nursing education may help narrow the gap between clinical practice as students and professional practice after graduation, thereby facilitating smoother transitions into the clinical environment. Furthermore, strengthening students’ ability to deliver safe nursing care will ultimately contribute to improving the overall quality of healthcare.

## Figures and Tables

**Table 1:** Participant characteristics (*N* = 204).

Characteristics	Categories	*n* (%)	M ±SD
Gender	Female	164 (80.4)	
	Male	40 (19.6)	
Age (year)	≤21	69 (33.9)	23.40 ±5.10
	22	58 (28.4)	
	≥23	77 (37.7)	
Grade	3rd	67 (32.8)	
	4th	137 (67.2)	
Major satisfaction	Dissatisfied	19 (9.3)	
	Moderate	38 (18.6)	
	Satisfied	147 (72.1)	
Grade point average	<4.0	52 (25.5)	
	3.50~3.99	102 (50.0)	
	>3.50	50 (24.5)	
Clinical practice satisfaction	Dissatisfied	21 (10.3)	
	Moderate	46 (22.5)	
	Satisfied	137 (67.2)	
Experience of being injured during clinical practice	Yes	17 (8.3)	
	No	187 (91.7)	
Experience receiving blood and body fluid exposure during clinical practice	Yes	13 (6.4)	
	No	191 (93.6)	
Experience with Exposure Reporting	Yes	2 (1.0)	
	No	19 (9.3)	
	Not applicable	183 (89.7)	

**Table 2:** Descriptive statistics for study variables (*N* = 204).

Variables/Sub-Categories	Possible Range	Item Range	M ±SD
Nursing professionalism	1–5	1.5–5.00	3.87 ± 0.52
Standard precautions knowledge	0–29	13.00–29.00	25.40 ± 2.15
Moral courage	1–7	3.92–7.00	5.52 ± 0.63
Standard precautions performance	1–5	2.92–5.00	4.62 ± 0.39
Hand hygiene	1–5	2.71–5.00	4.55 ± 0.70
Personal protective equipment (PPE)	1–5	1.00–5.00	4.63 ± 0.59
Respiratory etiquette	1–5	2.67–5.00	4.63 ± 0.55
Treatment devices and supplies	1–5	2.50–5.00	4.80 ± 0.43
Environmental management	1–5	2.00–5.00	4.61 ± 0.59
Laundry management	1–5	2.00–5.00	4.66 ± 0.56
Safe injection practices	1–5	2.50–5.00	4.78 ± 0.49
Staff safety	1–5	3.33–5.00	4.90 ± 0.29

**Table 3:** Differences in standard precautions performance by Characteristics of Participants (*N* = 204).

Characteristics	Categories	*n* (%)	M ±SD	*t* or F (*p*) Post Hoc	*η^2^*
Gender	Female	164 (80.4)	4.65 ± 0.38	0.11 (0.915)	
	Male	40 (19.6)	4.61 ± 0.44		
Age (year)	≤21	69 (33.9)	4.62 ± 0.37	0.11 (0.696)	
	22	58 (28.4)	4.68 ± 0.39		
	≥23	77 (37.7)	4.65 ± 0.41		
Grade	3rd	67 (32.8)	4.62 ± 0.45	−0.81 (0.390)	
	4th	137 (67.2)	4.66 ± 0.36		
Major satisfaction	Dissatisfied ^a^	19 (9.3)	4.41 ± 0.43	5.35 (0.005) a < c	
	Moderate ^b^	38 (18.6)	4.58 ± 0.40		0.05
	Satisfied ^c^	147 (72.1)	4.70 ± 0.38		
Grade point average	<4.0 ^a^	52 (25.5)	4.60 ± 0.40	5.48 (0.005) c < b	
	3.50~3.99 ^b^	102 (50.0)	4.73 ± 0.33		0.06
	>3.50 ^c^	50 (24.5)	4.53 ± 0.46		
Clinical practice satisfaction	Dissatisfied	21 (10.3)	4.63 ± 0.38	2.28 (0.105)	
	Moderate	46 (22.5)	4.65 ± 0.40		
	Satisfied	137 (67.2)	4.69 ± 0.39		
Experience of being injured during clinical practice	Yes	17 (8.3)	4.69 ± 0.33	0.45 (0.652)	
	No	187 (91.7)	4.64 ± 0.40		
Experience receiving blood and body fluid exposure during clinical practice	Yes	13 (6.4)	4.59 ± 0.36	−0.58 (0.565)	
	No	191 (93.6)	4.65 ± 0.40		
Experience with Exposure Reporting	Yes	2 (1.0)	4.89 ± 0.15	0.52 (0.595)	
	No	19 (9.3)	4.60 ± 0.37		
	Not applicable	183 (89.7)	4.65 ± 0.40		

Note: Different lowercase letters (e.g., a, b, c) indicate statistically significant differences between groups based on Scheffé’s post hoc test (*p* < 0.05).

**Table 4:** Correlation among Variables (*N* = 204).

Variables	Nursing Professionalism	Standard Precautions Knowledge	Moral Courage	Standard Precautions Performance
	r (*p*)	r (*p*)	r (*p*)	r (*p*)
Nursing professionalism	1			
Standard precautions knowledge	0.168 (0.016)	1		
Moral courage	0.513 (<0.001)	0.195 (0.005)	1	
Standard precautions performance	0.306 (<0.001)	0.190 (0.006)	0.399 (<0.001)	1

**Table 5:** Factors affecting standard precautions performance (*N* = 204).

Variables	B	SE	β	t	*p*
(Constant)	3.27	0.22		14.82	<0.001
Moral courage	0.24	0.04	0.38	5.93	<0.001
GPA 3.50–3.99	0.14	0.05	0.18	2.73	0.007
F = 23.48(<0.001), R^2^ = 0.19, Adj.R^2^ = 0.18					

Dummy variables: Reference GPA < 3.50.

## Data Availability

The data presented in this study are available on request from the corresponding author due to ethical reasons.

## Abbreviations

The following abbreviations are used in this manuscript:

GPA	Grade Point Average
